# Eliminating Bad Debt Reimbursement to Hospitals Serving Traditional Medicare Beneficiaries

**DOI:** 10.1001/jamanetworkopen.2025.26402

**Published:** 2025-08-11

**Authors:** Jason D. Buxbaum, Ari D. Ne’eman, Cyrus M. Kosar

**Affiliations:** 1 Center for Advancing Health Policy through Research, Department of Health Services, Policy, and Practice, Brown University School of Public Health, Providence, Rhode Island; 2 Department of Health Policy and Management, Harvard T.H. Chan School of Public Health, Boston, Massachusetts; 3 Center for Gerontology and Healthcare Research, Department of Health Services, Policy, and Practice, Brown University School of Public Health, Providence, Rhode Island

## Abstract

This cross-sectional study reports the prevalence of and factors associated with bad debt eligible for Medicare reimbursement among US hospitals.

## Introduction

The Medicare program reimburses hospitals 65% of cost-sharing that Traditional Medicare (TM) beneficiaries fail to pay so long as the hospital makes reasonable efforts to collect the debt (42 CFR 413.89). Congress is considering a phase-out of bad debt reimbursement to reduce federal expenditures.^1^ However, the distributional consequences of eliminating hospital bad debt reimbursement—and savings that could be achieved—are inadequately understood.

## Methods

This cross-sectional study of general short-term hospitals was not human participants research as defined by 45 CFR 46; institutional review board approval was therefore not pursued. We studied general short-term hospitals with 2023 cost report data.^2,3,4^ We excluded hospitals with mean length of stay greater than 30 days, missing net patient revenue, or those located outside the 50 states or Washington, DC. We characterized hospitals as high bad debt if TM bad debt represented 0.5% or more of net patient revenue. We winsorized continuous variables by substituting the value at the 2.5 percentile (97.5 percentile) of the distribution for all values below the 2.5 percentile (97.5 percentile). We compared characteristics across high bad debt using mean differences for binary variables and standardized mean differences (SMDs) for continuous variables. Data analysis used R version 4.4 (R Project for Statistical Computing). We followed the STROBE reporting guideline.

## Results

Of 4293 study hospitals (Table and Figure), 3952 (92%) reported Medicare-eligible bad debt in 2023. Total Medicare bad debt was $2 549 796 622, or a mean (SD) of $593 942 ($906 811) per hospital (Table and Figure). There were 848 hospitals that reported bad debt of at least 0.5% of net patient revenues (Table and Figure). These high bad debt hospitals were smaller (mean [SD] beds, 64.7 [80.7] vs 164.1 [178.8] beds; SMD = −0.72) and had higher ratios of Medicare bad debt to beds (mean [SD], $15 558 [$17 104] vs $3176 [$3151]; SMD = 1.01) (Table). High-bad debt hospitals reported lower operating margins (mean [SD] −0.6 [14.3] vs 2.2 [13.8] points; SMD = −0.20), but similar liquidity (mean [SD] net asset ratio, 0.63 [0.77] vs 0.67 [0.79]; SMD = −0.05). Nearly three-fifths of high–bad debt hospitals were critical access hospitals (723 of 3445 hospitals [59%] vs 497 of 848 hospitals [21%]; mean difference, 0.38). While hospitals in California, Texas, and Florida collectively reported nearly $725 million in bad debt, hospitals with high bad debt were disproportionately located in the Upper Midwest (Figure).

**Table.** Hospital Characteristics Across High Bad Debt^a^

Characteristic	Hospitals, No. (%) (N = 4293)		Standardized mean difference^c^
	Without high bad debt (n = 3445)	With high bad debt (n = 848)^b^	
Bad debt			
Hospital, mean (SD), $ (thousands)	567.3 (907.7)	702.1 (895.5)	0.15
Per bed, mean (SD), $ (thousands)	3.2 (3.2)	15.6 (17.1)	1.01
Ownership			
Private	599 (17.4)	149 (17.6)	absolute percentage point difference, 0.00
Not-for-profit	2171 (63.0)	493 (58.1)	absolute percentage point difference, −0.05
Public	675 (19.6)	206 (24.3)	absolute percentage point difference, 0.05
Region			
Northeast	461 (13.4)	65 (7.7)	absolute percentage point difference, −0.06
West	689 (20.0)	162 (19.1)	absolute percentage point difference, −0.01
South	1286 (37.3)	330 (38.9)	absolute percentage point difference, 0.02
Midwest	1009 (29.3)	291 (34.3)	absolute percentage point difference, 0.05
Teaching	1219 (35.4)	127 (15.0)	absolute percentage point difference, −0.20
Utilization and capacity			
No. of inpatient days (thousands), mean (SD)	39.1 (51.6)	11.3 (18.9)	−0.71
No. of beds, mean (SD)	164.1 (178.8)	64.7 (80.7)	−0.72
Occupancy, mean (SD), %	48.3 (24.8)	37.7 (21.8)	−0.46
Income and prices			
Operating margin, mean (SD), points^d^	2.2 (13.8)	−0.6 (14.3)	−0.20
Commercial prices, mean (SD), % of Medicare	147.7 (45.6)	132.7 (45.2)	−0.33
Patient mix			
Length of stay, mean (SD), d	5.1 (2.4)	6.0 (3.4)	0.30
Medicaid days, mean (SD), %	18.7 (12.6)	14.3 (12.8)	−0.35
Balance sheet			
Net asset ratio, mean (SD)^e^	0.67 (0.79)	0.63 (0.77)	−0.05
Cash on hand, mean (SD), $ (thousands)	21.9 (53.8)	8.9 (23.6)	−0.31
Rural designation			
Rural facility qualification^f^	1396 (40.5)	633 (74.6)	absolute percentage point difference, 0.34
Critical access hospital	723 (21.0)	497 (58.6)	absolute percentage point difference, 0.38
Metropolitan area	2286 (66.4)	314 (37.0)	absolute percentage point difference, −0.29
System affiliation	2703 (78.5)	552 (65.1)	absolute percentage point difference, −0.13

^a^ Sources: Hospital cost reports,^2^ Centers for Medicare & Medicaid Services Open Payments Reports,^3^ and Agency for Healthcare Research and Quality Compendium of Health Systems.^4^

^b^ High–bad debt hospitals have Medicare-eligible bad debt ≥0.5% of net patient revenues for 2023.

^c^ Absolute percentage point difference for dichotomous and categorical variables (eg, ownership) and standardized mean difference for other characteristics. A standardized mean difference of 0.2 suggests a small difference, a standardized mean of 0.5 suggests a moderate difference, and an SMD of 0.8 suggests a large difference.

^d^ Operating margin is net income minus income from contributions, investments, and public appropriations divided by the sum of net patient revenue and other income except income from contributions, investments, and appropriations.

^e^ Net asset ratio is the difference between total assets and total liabilities, divided by total assets.

^f^ Rural facility qualification refers to hospitals with low-volume adjustment eligibility, critical access hospital type, sole community hospital designation, or Medicare dependent hospital designation.

**Figure.**
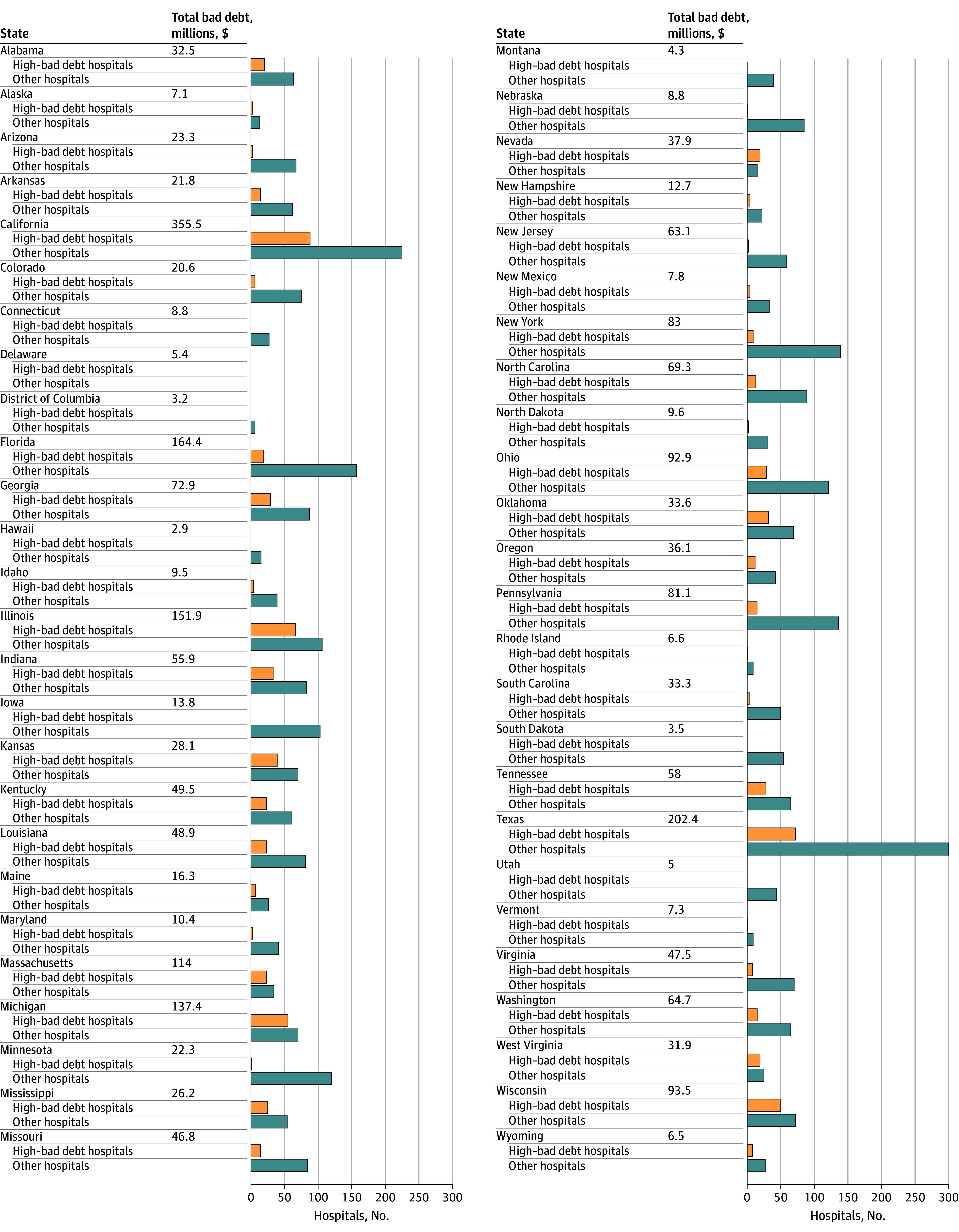
Medicare-Eligible Bad Debt Across States Total bad debt reported by in-state hospitals (millions of $) and count of hospitals with and without high bad debt, by state. Source: Hospital Cost Reports (2025).^2^

## Discussion

Under current law, 65% of the $2.6 billion in current TM hospital bad debt is eligible for reimbursement. The estimates from this cross-sectional study imply that this $1.7 billion in federal expenditures might be averted were bad debt reimbursement eliminated. Hospitals that would be most severely impacted by a phase-out of bad debt payments were geographically dispersed but disproportionately smaller and lower margin.

If Congress elects to phase-out bad debt reimbursement, high–bad debt hospitals might be supported through other policies. For instance, the Medicare Payment Advisory Commission has proposed consolidating existing hospital supports into a new composite Medicare Safety Net Index.^5^ Such an index might better target support to the most vulnerable hospitals—including those historically reliant on bad debt reimbursement—while reducing payments to more secure hospitals. Recognizing that the characteristics of a high bad debt hospital meaningfully differ from some other measures of safety-net status (eg, payer mix),^6^ our findings emphasize the merit of factoring in a range of facility characteristics in a Medicare Safety Net Index.

Limitations of this study include reliance on cost report data, which limited our ability to observe bad debt payments made by Medicare Advantage plans. Winsorizing extreme cost report values may have reduced noise at the expense of understating true differences across high bad debt. Furthermore, we did not consider bad debt payments to skilled nursing facilities and other Part A facilities. For this reason, our estimate of federal savings from a phase-out of bad debt reimbursement is lower than the $2.1 to $4.2 billion in savings forecast by the Congressional Budget Office.^6^ Additionally, we did not model how various other policies—such as changing criteria for Medicaid federal financial participation—might interact with changes to bad debt reimbursement. This is a critical area for analysis. Further scrutiny may be merited before Congress eliminates reimbursement for bad debt.

## Notes

Supplement.
Data Sharing Statement

## References

[1] United States House Committee on Ways and Means - Health budget options. Published January 2025. Accessed February 5, 2025. https://www.finance.senate.gov/imo/media/doc/budget_optionspdf.pdf

[2] RAND Corporation. RAND hospital data. Accessed July 10, 2025. https://www.hospitaldatasets.org/

[3] Centers for Medicare & Medicaid Services. Open payments list of teaching hospitals. Published October 2023. Accessed July 15, 2025. https://www.cms.gov/files/document/2024-reporting-cycle-teaching-hospital-list-oct2023x.xlsx

[4] Agency for Healthcare Research and Quality. Compendium of U.S. health systems, 2023. Published December 2024. Accessed July 15, 2025. https://www.ahrq.gov/chsp/data-resources/compendium-2023.html

[5] Medicare Payment Advisory Commission. Hospital inpatient and outpatient services: MedPAC Report to the Congress: Medicare Payment Policy. Published March 2023. Accessed February 5, 2025. https://www.medpac.gov/wp-content/uploads/2023/03/Ch3_Mar23_MedPAC_Report_To_Congress_SEC_v2.pdf

[6] Congressional Budget Office. Options for reducing the deficit: 2025 to 2034, mandatory spending, function 570, Medicare. Published December 12, 2024. Accessed February 5, 2025. https://www.cbo.gov/budget-options/60905

