# Supplementary material for: Eliminating Bad Debt Reimbursement to Hospitals Serving Traditional Medicare Beneficiaries
Source: JAMA Netw Open. 2025 Aug 11;8(8):e2526402. doi: 10.1001/jamanetworkopen.2025.26402 (PMC12340656; doi:10.1001/jamanetworkopen.2025.26402)
Supplement: Supplement. — Data Sharing Statement [file jamanetwopen-e2526402-s001.pdf]

Data Sharing Statement

Buxbaum. Eliminating Bad Debt Reimbursement to Hospitals Serving Traditional Medicare Beneficiaries. *JAMA Netw Open*. Published August 11, 2025. doi:10.1001/jamanetworkopen.2025.26402

Data
Data available: No

Additional Information
Explanation for why data not available: Data derived from (subscription) dataset prepared by RAND.
